# Juror decision-making and biracial targets

**DOI:** 10.3389/fcogn.2024.1354057

**Published:** 2024-04-18

**Authors:** Susan Yamamoto, Evelyn M. Maeder

**Affiliations:** ^1^Department of Psychology, Carleton University, Ottawa, ON, Canada; ^2^Department of Psychology, Campion College, University of Regina, Regina, SK, Canada; ^3^Institute of Criminology and Criminal Justice, Carleton University, Ottawa, ON, Canada

**Keywords:** biracial, essentialism, heuristics, juror decision-making, race salience

## Abstract

**Objectives:**

This study examined potential bias against Biracial defendants using a juror decision-making paradigm. We also tested whether encouraging mock jurors not to endorse racial essentialism (belief that racial groups have inborn, immutable traits that influence behavior) would mitigate bias.

**Methods:**

Canadian jury-eligible participants (*N* = 326) read a fabricated first-degree murder of a police officer case (involving a Black, White, or photo-morphed Black-White Biracial defendant), then made verdict decisions, completed a heuristics questionnaire, and answered racial categorization questions.

**Results:**

While there were no significant effects on verdicts, those higher in heuristic thinking tended to estimate a lower percentage of European ancestry for a Biracial defendant when the defense lawyer drew attention to race.

**Conclusions:**

Findings suggest that individual differences such as the tendency to rely on heuristic thinking may alter how racially ambiguous targets are perceived.

For decades psycho-legal researchers have studied racial discrimination in the criminal legal system. The topic remains relevant today, with minority groups overrepresented in prisons (Office of the Correctional Investigator, 2019). Two meta-analyses confirm that bias among jurors is one likely source of such inequality (Mitchell et al., 2005; Devine and Caughlin, 2014). However, race has primarily been conceptualized as a discrete, prototypical physical feature. In fact, the U.S. Census defined racial group memberships as mutually exclusive until 2000 (Chao et al., 2013). Consequently, there is a substantial gap in the literature regarding juror bias against racially ambiguous defendants. Trends suggest that the proportion of people identifying as multi-racial is growing (Young et al., 2017). Psycho-legal research should expand beyond fixed definitions of race to accommodate this reality.

Recent work also shows that a prominent bias reduction technique (i.e., making race a salient issue; Sommers and Ellsworth, 2001) can be ineffective and even detrimental for some groups (Maeder et al., 2015). It is therefore vital to identify intervention strategies that could be effective for a broader range of people and contexts. The purpose of this juror simulation study was to test whether an alternative strategy, which does not rely on a specific racial category, would reduce potential discriminatory decision-making in cases involving both racially prototypical and Biracial defendants.

## Racial categorization

Racial categorization is a complex process comprising both physical features like skin color (i.e., bottom-up processes) and high-order processes like prejudice (i.e., top-down processes; Freeman and Ambady, 2011). In an encounter, multiple sources are available to estimate group membership (e.g., gender, race, age) and sometimes cues are unclear (Bodenhausen and Peery, 2009). When cues are ambiguous, existing cognitive biases may exert a stronger influence. Pauker et al. (2009) conducted a series of experiments demonstrating that memory for biracial target faces (created using artificially but precisely generated computer images) varies as a function of motivation to include those targets as in-group members. Chen and Hamilton (2012) found that multiracial categorizations took more time and cognitive resources as compared to categorizing targets as monoracial. Bias may, therefore, interfere at the level of categorization, long before a formal judgment.

Stepanova and Strube (2012) found that for lighter skinned (but not darker skinned) targets, participants assess facial structure. Consequently, they argued that while prototypical racial stimuli (e.g., a person easily identifiable as Black) help in understanding stereotyping, targets with less group-typical features might be particularly vulnerable to individual differences in prejudice. Indeed, in their study of categorization of Black/White multiracial faces, Ho et al. (2015) found that people high in essentialism (i.e., the concept that races share an inborn “essence” that shapes their behavior) and anti-Black bias were more likely to categorize multiracial targets as Black rather than multiracial or White, in line with their negative attitudes. If some target races are categorized based on different cognitive processes, then these groups might also require different intervention strategies. Given the likely role of top-down processing in categorization of Biracial targets, the tendency to rely on mental shortcuts is one individual difference that may be demonstrative in examining characteristics of the juror that impact on decision-making.

## Race salience

Because people can be explicitly non-racist while still holding implicit racial biases, people might need reminding of their potential for engaging in discriminatory behavior when it conflicts with outward beliefs (Dovidio and Gaertner, 2000). One proposed way of combating this bias involves making jurors cognizant of their potential for discriminatory judgments by making race a salient issue (Sommers and Ellsworth, 2001). Bucolo and Cohn (2010) found that when an attorney explicitly argued that a defendant is only being charged because he is Black, jurors were less biased. However, in Canada, race salience manipulations have either no effect (Maeder et al., 2015) or increase bias (when participants first read a news article about racial bias; McManus et al., 2018).

## Racial certainty

As Sommers and Ellsworth (2009) noted, race salience theory is commonly misconstrued. The original concept referred to the prominence of themes of racism, not of the defendant's race *per se*. Traditionally, then, the technique has two components: racial certainty and prominence of racial issues, or as Bucolo and Cohn (2010) described it, the perceived “race card.” Sommers and Ellsworth (2009) argued that in real life, a defendant's race would be self-evident, but this is only true of racial groups for whom there are strong categorization cues. For instance, Indigenous persons are not identifiable by skin color; many people are also unfamiliar with stereotypic phenotypes or common names. After all, some groups' racialization depends upon characteristics of the perceiver just as much as the target, and Biracial individuals may identify with either, both, or neither group (Townsend et al., 2012).

The tendency to categorize Biracial targets as Black can be traced back to the “one-drop rule,” which maximized the number of individuals who could be subjugated. Formally termed hypodescent—categorization based on the most socially marginalized group—this phenomenon appears dependent on whether decision-makers believe race provides meaningful information about a person (Chao et al., 2013). Chen et al. (2014) showed that internal motivation to control prejudice predicts increased Biracial categorization. They further posited that motivation to externally appear unprejudiced may be associated with defaulting to monoracial categories. More recently researchers have found a minority categorization bias in line with hypodescent, but including categorizations other thank Black (Chen et al., 2018).

It is possible that drawing attention to a defendant's racialized status might encourage reliance on stereotypes, rendering the race salience technique problematic. It stands to reason that making participants cognizant of the lack of differences between racial groups could help protect against such stereotype-based judgments.

## The current study

If race is believed to yield meaningful inferences, then it is perceived as more relevant to a judgment (Chao et al., 2013). It might, therefore, be helpful to draw attention away from racial differences while encouraging bias monitoring. Moreover, it is unclear whether jurors would accept a race salience argument for a Biracial defendant, who might be subject to racial “gatekeeping.” If calling the defendant “Black” differs from participants' natural categorizations, it is possible that reactance would result (i.e., hostility toward the “race card”). One alternative intervention is to target the way that people think about race, by reducing the belief that race has an essence that informs behavior (i.e., racial essentialism; Ho et al., 2015). Inducing racial essentialism has been shown to increase the likelihood of categorizing targets based on race rather than other social identities (Chao et al., 2013), categorizing Biracial Black/White targets as Black (Ho et al., 2015), and sensitivity to phenotypic variations (Chao et al., 2013). In the current study, mock jurors read a fictional trial transcript in which the defendant's race varied (Black, White, Biracial). The defense lawyer's closing argument also varied, with one condition featuring a traditional race salience argument (Bucolo and Cohn, 2010), one featuring a statement aimed at discouraging racial essentialism, and one serving as a control with no such statement about race.

### Hypothesis 1: discrete categorization manipulation check

The first outcome of interest was participants' selection of a racial category for the target. We predicted a contingency between race salience and discrete racial categorization such that participants would be more likely to categorize a Biracial defendant as Black in the specific race salience condition as compared to the non-essentialism condition. Research shows that in situations of ambiguity, people are more likely to rely on biases (Dovidio and Gaertner, 2000). As such, drawing explicit attention to the negative effects of racial categorization could reduce the likelihood of hypodescent.

### Exploratory analysis: continuous categorization

The second major outcome of interest was participants' estimation of amount of European ancestry on a continuous scale, for which we did not form a prediction. However, previous research suggests that internal motivation to control prejudice increases recognition of Biracial categories while external motivation may instead relate to discrete categorization (Chen et al., 2014). Drawing attention to racial categorization as a source of prejudice could decrease reliance on discrete group identification.

### Hypothesis 2: verdict decision

Research suggests that people with both Black and White ancestry are more likely to be categorized as Black (Chao et al., 2013). As such, we predicted that both the Black and Biracial defendants would receive a significantly higher proportion of guilty verdicts as compared to the White defendant. In line with race salience theory, we expected this main effect to be qualified by an interaction, such that the effect would disappear in conditions in which race is made salient (both through racial essentialism and the traditional manipulation). Although the race salience technique can be ineffective in a Canadian context, this backfire effect is more consistent for Indigenous targets, which were not included in this study. Our hypothesis therefore followed the classic U.S. based aversive racism literature. We expected heuristics to moderate this effect, such that the non-essentialism manipulation would mitigate bias for those higher on the trait.

## Method

### Participants

The initial sample^1^ consisted of 397 Canadian jury-eligible (citizens at least 18 years of age having no indictable offenses without formal pardon and not having an excludable occupation) Mechanical Turk (MTurk) workers. Those who completed the survey in under 10 min (approximately half of the median time of 21 min) were removed prior to analyses, resulting in 326 participants (177 men, 145 women, 2 non-binary individuals, 1 transgender individual, 2 unspecified), whose ages ranged from 18 to 68 (*M* = 33.6, *SD* = 10.5). A majority of the sample identified as White (72.7%), with 4.3% identifying as Black, and roughly 3% as an unlisted race. A table with the self-identified ethnic background of the sample can be found in the Supplementary material on Open Science Framework (OSF).

### Materials/procedure

This study was cleared by the university's Research Ethics Board. Interested participants accessed a link to Qualtrics survey software via an ad on MTurk and completed juror eligibility screening. After reading instructions on the charge, self-defense, the burden of proof, and reasonable doubt (National Judicial Institute, 2014), participants read a 15-page fabricated trial transcript featuring a defendant charged with first-degree murder of a police officer, who pleads self-defense (loosely based on R. v. Gayle, [2001]). They then made a dichotomous verdict decision.

Race was manipulated via color photographs of White and Black persons (one for each racial group), which were pilot tested (*N* = 30) to ensure correct racial identification and to match on perceived age, attractiveness, and likeability. Following previous work (Ho et al., 2015) we merged the two photographs to form one multi-racial face (Black/White) using photo morphing software (Morpheus Software, 3.17, Morpheus Development LLC, Howell, MI). For the race salience manipulation, the defense lawyer made a brief argument during closing statements. The manipulation featured three levels: race not salient (control), specific race salient (“We all know that race is the only reason we're here today. My client would never have been charged if he were not Black”), and non-essentialism salient (“We all know that racism is why we're here today but remember that race is a made-up concept. We share 99.9% of our genetic makeup—race can't tell us about someone's character”). These manipulations resulted in a 2 (Black, Biracial) by 3 (control, specific race salient, non-essentialism) between-subjects factorial design, with one floating cell for a White defendant control condition.

Participants then completed a 15-item heuristics questionnaire (e.g., “It seems natural to use red in a traffic light to mean stop;” Salomon and Cimpian, 2014), which showed strong internal consistency (α = 0.83). Next, participants made a discrete categorization (i.e., “What was the race of the defendant?”) choosing from Asian, Black, East Indian, Hispanic/Latino, Indigenous Peoples, Middle Eastern, White, and Not listed (please specify). Additionally, they responded to five continuous measures: an estimated percentage of Black, European, Asian, Latino, and Indigenous ancestry. Finally, participants completed a demographics questionnaire. The study took approximately 20 min and participants were compensated with $1.50.

## Results

### Hypothesis 1: discrete categorization

Results of a Chi-square contingency test supported the prediction that participants would be more likely to categorize the Biracial defendant as Black in the specific race salience condition. In the control condition, participants were equally likely to report that the defendant was White (41.4%) or Black (40.6%). Most participants reported that the defendant was Black in the non-essentialism condition (67.3%), with the highest count in the specific race salient condition (81.3%). Table 1 displays the categorization breakdown by condition along with adjusted standardized residuals.

**Table 1:** Biracial defendant categorization breakdown by condition.

	**Other**	**White**	**Black**
Control	*n* = 23	*n* = 53	*n* = 52
	18.0%	41.4%	40.6%
	*z* = 0.80	*z* = 6.2	*z* = −6.0
Essentialism	*n* = 20	*n* = 15	*n* = 72
	18.7%	14.0%	67.3%
	*z* = 0.90	*z* = −2.8	*z* = 1.7
Specific	*n* = 9	*n* = 8	*n* = 74
	9.9%	8.8%	81.3%
	*z* = −1.9	*z* = −3.9	*z* = 4.7

### Exploratory analysis: continuous categorization

As an exploratory analysis, we used Hayes Process Macro (Hayes, 2022) with race salience as the independent variable, heuristics as a moderator, and European ancestry as the dependent variable (for the Biracial defendant condition). There was a significant heuristics by race salience interaction effect. Using the control condition as the referent, the non-essentialism effect was non-significant. However, the specific race salience by heuristics interaction effect was significant, *t* = −34.35, *SE* = 12.43 *p* = 0.008. Teasing apart the interaction, it seems that the difference for those at low (*M* = 2.73) and moderate (*M* = 3.40) levels of heuristics was non-significant, whereas the difference was significant for those at high levels (*M* = 4.07, *t* = −35.13 *SE* = 11.38, *p* = 0.003, 95% CI: [−57.93, −12.32]). Thus, it seems that only those high in heuristic thinking tended to estimate a lower percentage of European ancestry for a Biracial defendant when the defense lawyer drew attention to his specific race.

### Hypothesis 2: verdict decision

Table 2 displays the verdict counts for each experimental condition. A chi-square contingency test revealed that, in the control conditions, there were no significant differences in verdict distribution as a function of defendant race, $\chi_{\left(2\right)}^{2}$ = 0.90, *p* = 0.64, Φ = 0.08. All three conditions were heavily in favor of a not guilty verdict.

**Table 2:** Verdict breakdown for all conditions.

		**Not guilty**	**Guilty**
Control	White	32	16
		66.7%	33.3%
	Biracial	31	12
		72.1%	27.9%
	Black	23	14
		62.2%	37.8%
Non-essentialism	Biracial	34	22
		60.7%	39.3%
	Black	37	14
		72.5%	27.5%
Specific	Biracial	35	15
		70.0%	30.0%
	Black	24	17
		58.5%	41.5%

To test the hypothesis that the non-essentialism salient condition would produce a lower likelihood of a guilty verdict for the Biracial defendant for those high in heuristic thinking, we conducted a hierarchical binary logistic regression using race salience dummy codes (with the control condition as the referent) and mean score on heuristic thinking as the independent variables and verdict decision as the dependent variable. There was a significant main effect of heuristics, such that a higher score was associated with an increased likelihood of a guilty verdict, *B* = 0.95, *SE* = 0.34, *p* = 0.005, *exp(B)* = 2.58. There were no other significant effects. This analysis was repeated to compare the essentialism and specific conditions, with no other significant effects. A hierarchical binary logistic regression probing a three-way interaction between race (Black, Biracial), race salience, and heuristics again yielded only a significant effect of heuristics (see OSF page for regression table).

## Discussion

The purpose of this study was to examine the treatment of Biracial targets in a juror simulation paradigm. Given that Biracial individuals may fall into either racial category, we also tested an intervention strategy that does not draw attention to a singular group. Interestingly, participants in the control condition were fairly even split with respect to categorizing the Biracial defendant as Black or White, with a minority using the “unlisted” category. Conversely, the phenomenon of hypodescent would suggest a greater proportion of Black categorizations. Results revealed a greater proportion of Black categorizations only when the defense lawyer mentioned race generally or explicitly referred to the defendant as Black.

Results did not support the prediction that Black and Biracial targets would elicit a greater proportion of guilty verdicts as compared to a White defendant. There were also no significant verdict differences as a function of our salience manipulations. Although a rich history of experimental studies has demonstrated significant effects of race on juror decision-making, this trend may be changing. Recent work has failed to show the expected racial bias in verdict decisions, and we argue it is unlikely that this null effect is due primarily to actual reduced levels of racial bias. It could instead be that, given the prominence of racial injustices in the media, race is generally more salient. This may result in participants' desire to appear non-biased, which is especially likely for populations who have considerable experience with psychological studies (i.e., MTurk workers). There was a significant main effect of heuristics on verdict decisions, such that those higher in heuristic thinking had an increased likelihood of a guilty verdict. This finding could reflect greater reliance on extra-legal variables.

It is still unclear whether such effects represent a true decrease in bias or simply increased socially desirable responding. Indeed, external motivation to control prejudice, especially in situations of ambiguity, could result in greater likelihood of monoracial categorization (Chen et al., 2014). In the context of race salience, categorizing a defendant as Black aligns with the defense lawyer's attempt to reduce prejudice. Contrary to our intended effect, it is possible that the essentialism manipulation produced reactance—some people might interpret the assertion that “race is a made-up concept” as hostile, increasing rather than decreasing reliance on essentialism, rendering it ineffective. Future researchers may wish to compare internal and external motivation to control prejudice alongside other essentialism manipulations.

Part of the rationale behind this study was to more closely investigate perceptions of targets for whom bottom-up cues for categorization are more subtle. Inclusion of a Biracial target can help to uncover higher order cognitive biases that may determine how they are categorized. Previous work suggests that endorsing racial essentialism increases likelihood of perceiving the target as Black (Chao et al., 2013). In the current study, there was a significant race salience by heuristics interaction effect on our continuous measure of estimated ancestry. There were differences between race salience conditions, but only for those high in heuristics. For the Biracial defendant, there was a lower estimated percentage of European ancestry in the specific race salience condition as compared to the control condition. This finding suggests that the specific race salience manipulation only led a subset of participants to racialize the defendant. Individual differences in heuristic processing are therefore a potential variable to better understand receptivity to a race salience argument. Participants could have received the lawyer's traditional race salience argument in two ways. First, they might have deferred to his authority without further considering the claim. Second, they might have felt that the argument simply did not apply to the Biracial defendant. The data suggest the former may be the case for those high in heuristic thinking.

### Implications

One implication of our findings is that studying groups for whom skin color is not a strong cue to categorization poses unique challenges. Moreover, elimination of participants who fail discrete manipulation checks may obscure an important piece of the puzzle. Cognitive processes can significantly influence how a target is perceived, regardless of their “actual” race. An added challenge to this research paradigm is that there is no ground truth *per se* for a person's racial categorization, making it difficult to determine what would constitute a “wrong” answer. Nonetheless, researchers should consider analyzing data from failed manipulation checks in race studies to see if individual differences may be responsible. In a real-world setting, jurors will not always categorize a defendant in a uniform manner.

Research on racial essentialism implies that encouraging categorization based on race can have adverse effects. It is likely that a traditional race salience manipulation will be effective for some individuals and backfire for others (see McManus et al., 2018). More work is needed to ascertain the conditions under which it may be undesirable to invoke issues of race. Of equal importance, modern racial bias research may need to pivot to address the null effects of race on verdict decisions. It could be that social desirability is implicated, and it is unclear whether this would translate to actual motivation to make fair decisions in a courtroom setting.

### Limitations

There are some notable caveats to bear in mind. Foremost, researchers should test this paradigm using different recruitment methods (i.e., in-person, other online platforms). MTurk workers often have experience with racial bias studies, which may itself be a race salience effect (i.e., participants do not wish to appear prejudiced), and they may be distinct demographically (Paolacci et al., 2010). Future studies should also employ stimulus sampling. Moreover, courts may be skeptical of online data in general. Second, we did not control for juror race in the current study, which likely affects categorization. It could be that Biracial individuals are more likely to recognize other multi-racial persons. However, our participants were predominantly White, and this population is more at-risk for demonstrating bias against racialized defendants. Finally, it is also worth noting that the case-type itself, while a rare occurrence, is also prominent in public discourse. As such we cannot discount the possibility that the trial stimulus generally elicited a race salience effect.

## Conclusion

Findings of this study call for researchers to consider a wider range of targets in studies of racial bias. Individual differences such as the tendency to rely on heuristic thinking may alter how racially ambiguous targets are perceived, which has implications for the utility of traditional manipulation checks. It is also necessary to expand investigations of the traditional race salience manipulation to encompass more target groups.

## Data availability statement

The datasets presented in this study can be found in online repositories. The names of the repository/repositories and accession number(s) can be found below: https://osf.io/d7hzt/?view_only=61b9e796108440be94e7a02baab840a6.

## Ethics statement

The studies involving humans were approved by Carleton University Research Ethics Board. The studies were conducted in accordance with the local legislation and institutional requirements. The participants provided their written informed consent to participate in this study.

## Author contributions

SY: Conceptualization, Data curation, Formal analysis, Funding acquisition, Methodology, Writing – original draft. EM: Conceptualization, Methodology, Resources, Supervision, Writing – review & editing.

## Footnotes

^1^Raw data and supplementary material are available on Open Science Framework at https://osf.io/d7hzt/?view_only=61b9e796108440be94e7a02baab840a6.
